# Basic Cells for Reconfigurable Superconducting Kinemonics

**DOI:** 10.3390/nano16150940

**Published:** 2026-07-30

**Authors:** Anastasia A. Maksimovskaya, Vsevolod I. Ruzhickiy, Sergey V. Bakurskiy, Andrey E. Schegolev, Maxim V. Tereshonok, Nikolay V. Klenov, Igor I. Soloviev

**Affiliations:** 1 Faculty of Physics, Lomonosov Moscow State University, 119991 Moscow, Russia; stasyahime@gmail.com; 2 All-Russian Research Institute of Automatics n.a. N.L. Dukhov (VNIIA), 127055 Moscow, Russia; vi.ruzhickiy@physics.msu.ru (V.I.R.); r4zz@mail.ru (S.V.B.); igor.soloviev@gmail.com (I.I.S.); 3 Research Department, Moscow Technical University of Communications and Informatics (MTUCI), 111024 Moscow, Russia; a.e.shegolev@mtuci.ru (A.E.S.); n.v.klenov@mtuci.ru (N.V.K.); 4 Skobeltsyn Institute of Nuclear Physics, Lomonosov Moscow State University, 119991 Moscow, Russia

**Keywords:** superconducting electronics, Josephson junctions, tunable kinetic inductors, universal programmable logic cell, all-JJ logic cells, low power consumption

## Abstract

In all-Josephson-junction (all-JJ) logic, cell area is determined by the size of Josephson junctions, enabling intrinsically compact layouts. Tunable kinetic inductance offers a route to add circuit reconfigurability, pushing further scaling within the same all-JJ framework. In this work we present a set of basic cells for reconfigurable superconducting “kinemonics” that exploit tunable kinetic inductances of a multilayer nanostructure to realise multiple logic functions within a single compact circuit. We then combine these gates into a universal programmable logic cell consisting of only four reconfigurable gates supplemented by a single tunable kinetic-inductance key and demonstrate that it can realise all sixteen two-input Boolean functions, making it an analogue of a look-up table with in-hardware reconfigurability. We also discuss how the same principle can be used in superconducting neuromorphic circuits, where tunable kinetic inductance controls routing, coincidence detection, inhibition, and delay for soliton-like spikes in neuron-like elements.

## 1. Introduction

Rapid Single-Flux Quantum (RSFQ) logic, based on the Josephson effect and magnetic flux quantization, has long been a central superconductor digital technology, combining picosecond-scale switching with very low energy dissipation [1,2,3]. As circuit complexity and integration density increase, however, the physical size of both active and passive elements becomes a major scaling constraint. In particular, inductors occupy a substantial fraction of the chip area and limit the functional density of RSFQ circuits [4].

Kinetic inductance offers a direct physical route to reducing this area penalty. In thin or nanostructured superconductors, a substantial part of the inductive energy is stored in the inertia of the superconducting condensate [5] rather than in the magnetic field surrounding the conductor [6,7,8]. Therefore, narrow superconducting segments can provide large inductance per unit length and replace much larger geometric inductors. More broadly, kinetic inductance has become an active design resource in superconducting resonators, detectors, sensors, and quantum-circuit elements, rather than only a parasitic contribution to be minimized [9,10,11].

Beyond enabling circuit miniaturization, kinetic inductance provides additional functionality. Since it depends on the density of superconducting carriers, it can be sensitive to external stimuli and used as a functional, and in some cases tunable, circuit parameter. This principle underlies several established classes of devices. In photon and particle detectors, from microwave kinetic inductance detectors to superconducting single-photon detectors, the pair-breaking response of a thin superconducting film converts the absorbed energy into a measurable inductance change [12,13]. A second class of devices exploits the current nonlinearity of the kinetic inductance that develops as the bias approaches the depairing current; this regime underlies kinetic-inductance parametric up-converters, magnetometers, current sensors and non-linear resonators [14,15,16,17,18]. Its most prominent present-day representative is the kinetic-inductance travelling-wave parametric amplifier (KI-TWPA), which provides broadband, near-quantum-limited microwave amplification for qubit and cryogenic-detector readout [19,20,21,22,23]. A third group of applications uses the kinetic inductance as an in situ adjustable parameter: dc-current-tuned resonators made of high-resistivity films [24,25]. Finally, kinetic inductance serves as a compact substitute for geometric inductance in nanoscale memory nanowire loops [26] and elements of quantum circuits [27]. For superconducting computing systems, both classical and quantum, compact kinetic-inductance elements can increase integration density and reduce parasitic magnetic crosstalk by replacing large inductive loops with local nanoscale structures [28].

In this work, the term “superconducting kinemonics” denotes a motion-based circuit architecture rather than a particular device. Information is represented and processed by the controlled motion of spatially localized phase, current, and voltage excitations through a superconducting network. In a Josephson transmission structure, such an excitation is a propagating 2π phase-transfer event accompanied by an SFQ-like voltage pulse and a transient current wave; in our earlier terminology it is a kinetic soliton. Because the finite lumped all-JJ circuits considered here are not an integrable continuous medium, we use the more cautious expression “soliton-like excitation” when discussing the present cells.

Operationally, we call a circuit kinemonic when (i) information is carried by the motion of these localized excitations and (ii) the circuit function is realized by controlling their transmission, blocking, splitting, delay, or interaction. Tunable kinetic inductance is used sparsely, in a small number of key branches, where it both reduces the footprint relative to a geometric inductor and changes the trajectories or switching thresholds of the propagating excitations. Thus, “kinemonics” is not a synonym for all kinetic-inductance electronics. It is also distinct from the “kinemon” artificial atom, which is an inductively shunted transmon [27]; the latter is a specific quantum circuit element, whereas kinemonics here denotes an architecture for processing propagating excitations.

The use of kinetic inductors, pioneered as a route to compact superconducting digital circuits, addresses the inductor-area bottleneck but also introduces practical constraints. High-kinetic-inductance elements require tight control of film thickness, material composition or disorder, linewidth, edge quality, interface transparency, and critical-current margins, because all these factors affect the target value and spread of $L_{k}$. Our approach therefore treats kinetic inductance not as a universal replacement for every inductive element, but as a compact control key used where tunability creates additional functionality. The cells studied here are built within the all-Josephson-junction (all-JJ) logic framework, in which every geometric inductor is replaced by a Josephson junction, so that pulse propagation and logic operation are governed by Josephson phase dynamics and large passive inductive loops are avoided. Compact all-JJ logic has been developed along several lines, including circuits based on bistable Josephson junctions [29], logic built on 2φ-junctions driven by half-flux-quantum pulses [30], and 0−0−π inductorless blocks [31]. The elementary gates considered here fit naturally into this framework: the AND, OR and XOR operations are realised with conventional junctions having a sinusoidal current-phase relation and do not themselves require bistable elements. What we add to the all-JJ picture is reconfigurability. A single tunable inductive element, hereafter the kinetic-inductance control key (KICK) [32], is inserted into a small number of key branches; switching its effective inductance changes the dynamical conditions for pulse transmission and thereby selects which function a fixed junction network performs, without redesigning the junction layout. Fixed inductive functions are avoided by relying on all-JJ cell dynamics, while the tunable kinetic-inductance elements are reserved for mode selection and reconfiguration.

A possible physical implementation of such a tunable element is provided by superconducting spin-valve structures, where the relative orientation of ferromagnetic layers modifies the superconducting proximity state and can therefore change the kinetic inductance [33,34]. In circuit terms, this element acts as a KICK: by switching its inductive state, the same physical cell can be assigned different logical functions. This reconfigurability increases not only the density of individual elements, but also the functional density of the whole superconducting processor, because fewer distinct circuit blocks are needed to implement a larger set of operations. It also provides a mechanism for compensating fabrication spread and adjusting operating points without redesigning the full circuit. Notably, this control principle has recently been verified experimentally: in split-ring resonators fabricated from Nb/Co/Nb/Co/Nb/Al spin-trigger multilayers, remagnetisation of the ferromagnetic layers produced a reproducible, non-volatile shift of the resonant frequency at zero applied magnetic field, corresponding to a change in the kinetic inductance of the structure [35].

Here we present a set of basic cells for reconfigurable superconducting kinemonics. The library includes a controllable splitter, reconfigurable AND/OR and XOR/OR cells, and a universal programmable logic cell constructed from four reconfigurable gates and one tunable kinetic-inductance element. This universal programmable logic cell implements all sixteen two-input Boolean functions and therefore plays a role analogous to a look-up table, but with the functionality encoded in a configurable hardware basis rather than in a stored truth table.

The paper is organized as follows. Section 2 introduces the normalized Josephson-junction model and the simulation criteria used throughout the work. Section 3 presents the elementary kinetic-inductance-controlled cells: the controllable splitter, the AND/OR gate, and the XOR/OR gate. It also discusses the physical implementation of the KICK element and the associated practical constraints. Section 4 combines these gates into a universal programmable logic cell and discusses its interpretation as a compact reconfigurable Boolean block. Section 5 explains how the same programmable primitives can be interpreted in bio-inspired spiking circuits, where Boolean functions correspond to local operations such as coincidence detection, inhibition, gating, and routing. The influence of tunable kinetic inductance on the soma dynamics of a superconducting spiking neuron is also analysed there. Section 6 summarises the implications of the proposed approach for compact reconfigurable digital logic and neuromorphic superconducting hardware.

## 2. Methodology of Modelling

The nonlinear dynamics of the proposed cells is described by the resistively and capacitively shunted Josephson-junction (RCSJ) model [1]. For a conventional Josephson 0-junction, the current–phase relation below the critical current is $I_{s}=I_{c}\sin\varphi$, and the total current through the junction is expressed as

(1) $$I=I_{c}\sin\varphi +\frac{V}{R_{N}}+C\frac{dV}{dt},$$

Here, $R_{N}$ is the normal resistance, and *C* is the capacitance of the Josephson junction. All currents are normalized to a common reference critical current $I_{c,\mathrm{ref}}$ and all inductances to the corresponding Josephson inductance $L_{J}=\Phi_{0}/\left(2\pi I_{c,\mathrm{ref}}\right)$. This reference is a fixed normalization scale chosen once for the whole circuit; each junction is then characterized by a dimensionless amplitude $A=I_{c}/I_{c,\mathrm{ref}}$, which differs from unity when the junction’s critical current differs from the reference. In the neuron subsection a specific junction ($I_{c3}$) is taken as the practical reference. Time is normalized to the inverse plasma frequency $\omega_{p}=\sqrt{2\pi I_{c,\mathrm{ref}}/\left(\Phi_{0}C_{\mathrm{ref}}\right)}$, and Equation (1) takes the form

(2) $$i=A\sin\left(\varphi \right)+\alpha \dot{\varphi }+\beta \ddot{\varphi }.$$

Here, dots indicate derivatives with respect to time, *t*, normalized to the inverse plasma frequency, $\dot{\varphi }=2\pi V/\left(\Phi_{0}\omega_{p}\right)=V/V_{0}$, $\alpha =\Phi_{0}\omega_{p}/\left(2\pi I_{c,\mathrm{ref}}R_{N}\right)$ is a damping coefficient, $A=\pm I_{c}/I_{c,\mathrm{ref}}$, and $\beta =C/C_{\mathrm{ref}}$ is the ratio of the junction capacitance to the reference $C_{\mathrm{ref}}$. In the cells considered here all junctions share the same β, so this ratio carries no independent role and is kept only to make the normalization explicit.

Accordingly, the tunable kinetic inductance is expressed throughout in these normalized units, as the dimensionless ratio $L_{k}/L_{J}$, and it is this branch inductance that enters the circuit equations. Changing it modifies the phase–current relation of the corresponding branch and hence the redistribution of current between the Josephson junctions during pulse propagation, but it does not alter the intrinsic current–phase relation of any junction, which in the present model remains sinusoidal.

The ratio $L_{k}/L_{J}$ introduced above is formally analogous to the dimensionless screening parameter of a SQUID loop, $\beta_{L}=2\pi L_{\mathrm{loop}}I_{c}/\Phi_{0}$, where $L_{\mathrm{loop}}$ and $I_{c}$ denote the loop inductance and the junction critical current of the SQUID: the two expressions coincide in form once the same critical-current scale is used for normalization. In a conventional rf- or dc-SQUID, increasing this parameter can lead to multiple dynamically accessible states and to a hysteretic response, with the detailed behaviour depending also on damping, bias and drive conditions [36,37,38]. The circuits considered here, however, are multi-junction networks in which the kinetic inductor does not close a single-junction loop, and they are driven by finite pulse sequences rather than by a slowly swept flux; they therefore cannot be reduced to a single rf-SQUID, and no universal critical value of $L_{k}/L_{J}$ is implied. The onset of retained or history-dependent states is determined here by the full circuit topology and by the operating protocol, and the pulse-train criterion introduced below is designed specifically to reject such parameter regions from the memoryless operating domains.

In all simulations, a logical ‘1’ is represented by the arrival of a soliton-like voltage pulse at the corresponding input or output port, whereas logical ‘0’ corresponds to the absence of such a pulse within the prescribed time window.

The circuit equations, assembled from Equation (2) for every junction together with the node current-conservation relations, are integrated with an explicit Runge–Kutta method of the Dormand–Prince type with adaptive error control (relative and absolute tolerances $10^{-7}$ and $10^{-9}$). Each logical “1” is generated by exciting the input all-JJTL with a Gaussian current pulse of half-width $T_{0}=20$, which launches a single SFQ-like 2π phase-transfer event; every input pattern is applied as a train of three such pulses separated by $T_{\mathrm{per}}=250$, a period long enough for the transient to decay completely between events. The output is classified over a detection window centred on each expected arrival, and the remainder of the inter-pulse interval serves as the relaxation window, in which no further switching is permitted. The parameter maps were obtained on a uniform grid of step $\Delta (L_{k}/L_{J})=0.01$ over the ranges shown in the figures. Numerical convergence was verified by repeating representative sweeps with the tolerances tightened by an order of magnitude and the sampling step halved, which left the classified truth tables, and hence the domain boundaries, unchanged.

A cell is considered to operate correctly if, for each input pattern, the output line produces the pulse sequence required by the target truth table and no additional switching events occur after the transient relaxation time. Two further conditions are imposed. First, the output is classified through the transferred flux rather than a voltage threshold: the number of quanta delivered to a given line is obtained from the accumulated 2π phase slips of the corresponding junction ($\int V\;dt=\Phi_{0}$ per event) and must equal the expected count on every pulse of the train, exactly zero being required on every blocked line; the input patterns are additionally applied in different orders within the train, with the response required to be identical in each ordering. Second, at the end of the relaxation window the circuit is required to have reached a stationary state carrying the same static current distribution as before the event: the junctions that transmitted a quantum have advanced their phase by 2π, while the remaining phases have returned to their static bias values, so that no residual circulating current is carried over to the next event.

This protocol is deliberately stricter than a check of the output for an isolated event: at sufficiently large kinetic inductances a branch can support several stable states [32], and a retained state would bias the response to the following event, so that the cell would no longer be a memoryless Boolean gate. Applied simultaneously to all relevant input combinations, the test detects leakage into a blocked line, cross-talk between lines, and accumulation of current in the kinetic inductances from one pulse to the next, so that parameter regions in which the circuit retains a state, switches spontaneously, or responds in a history-dependent manner are excluded from the operational domains.

## 3. Reconfigurable All-Josephson Junction Logic Cells

The elementary cells considered below are realised within the all-JJ framework introduced in Section 1, with reconfigurability provided by the KICK elements. We first specify how this element is treated at the circuit level and how its states are mapped onto the operating modes of the individual cells.

In the simulations below, the KICK is treated as a tunable inductive branch with a discrete set of effective values. Microscopically, these values can be associated with different magnetic states of the multilayer superconducting heterostructure [39,40,41,42,43], such as a spin-valve-type element, in which the proximity-controlled superfluid density changes $L_{k}$ [33,34]. Since $L_{k}\propto n_{s}^{-1}$, switching the magnetic state corresponds to switching the effective inductive state. We use this mapping at the circuit level: $L_{\mathrm{on}}$ and $L_{\mathrm{off}}$ define the pass and block regimes of the splitter, whereas other selected values of $L_{k}$ set the operating mode of the AND/OR and XOR/OR cells.

A practical advantage of selecting the operating mode through kinetic inductance, rather than through a bias current, is the simplicity of control distribution. Bias-current selection would require a dedicated current line routed to a specific internal node of every gate, so that the wiring complexity and on-chip area overhead grow rapidly with the number of gates; the KICK avoids this and preserves the compactness of the all-JJ layout.

### 3.1. Physical Implementation of the KICK Element

Since the proposed logic concept rests on the availability of a tunable kinetic-inductance element, we briefly outline how such an element can be realised in practice. Several experimentally demonstrated mechanisms provide this functionality. The current dependence of the nonlinear kinetic inductance of high-resistivity superconducting films has been used to tune microstrip resonators over a wide frequency range [24,25,44]. An alternative route, which we consider the most natural for the KICK because it combines non-volatility with the absence of static control lines, is the magnetic control of the kinetic inductance in superconductor/ferromagnet multilayers [33,34,35].

In this approach the KICK is a spin-trigger multilayer of the SF_1_S_1_F_2_sN type. A thick superconducting layer S serves as a reservoir of Cooper pairs, the F_1_S_1_F_2_ spin valve transmits or suppresses pair correlations depending on the mutual orientation of the ferromagnetic magnetizations, and a thin trigger layer s together with a low-resistivity normal layer N forms a proximized low-inductance channel. Remagnetisation of the valve redistributes the supercurrent between the S electrode and the sN bilayer and thereby switches the kinetic inductance of the element between distinct non-volatile states [34]. Experimentally, such a stack has been realised as a Nb/Co/Nb/Co/Nb/Al multilayer [35]; on a chip the KICK is a narrow strip of this multilayer with a length of the order of 100–1000 nm placed between wide superconducting electrodes, so that its footprint is compatible with the all-JJ layout.

Microscopic calculations predict that remagnetisation changes $L_{k}$ of an optimized structure by several times, provided that the trigger-layer thickness lies close to its critical value, while the normal overlayer enhances the contrast between the parallel and antiparallel states by a factor of three to four [34]. The first experimental verification of this mechanism in split-ring resonators demonstrated a reproducible, non-volatile shift of the resonant frequency of about 4 MHz at zero applied field and confirmed the predicted enhancement by the aluminium overlayer [35]. Although the observed shift is reproducible and qualitatively consistent with the theoretical description, its magnitude is considerably smaller than the theoretically expected one, which is most likely related to the domain structure of the ferromagnetic layers.

The main practical constraints of the magnetic implementation are the following. The element remains a linear reactive component only at currents well below the depairing current of the multilayer [34]. Reaching the maximal contrast requires nanometre-scale control of the layer thicknesses, since the trigger layer must stay near its critical value. Finally, reprogramming relies on remagnetisation by local millitesla-scale field pulses and is limited to a nanosecond time scale, so the KICK is intended for configuration-type switching rather than for switching within every clock cycle. We note that the non-volatile magnetic switching of the kinetic inductance has been demonstrated experimentally [35], whereas the several-fold tuning contrast assumed for the splitter is at present a theoretical prediction for optimized structures. The main outstanding task is therefore further investigation of the tunable elements aimed at increasing the magnitude of the switching effect; the solution most likely lies in improving the quality of the ferromagnetic films and in the choice of the optimal ferromagnetic material for the spin-trigger structure [33].

### 3.2. Splitter

**Figure 1:**
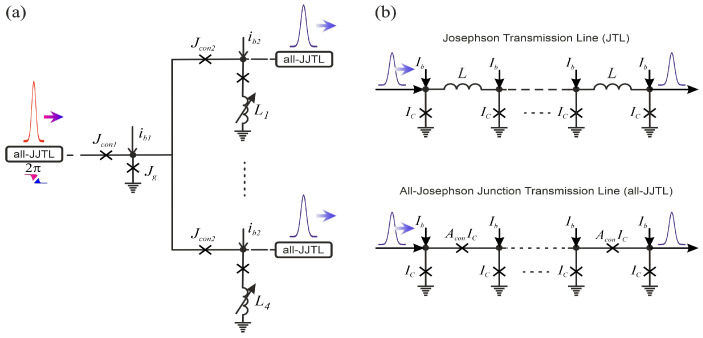
(**a**) Splitter circuit. (**b**) Conventional JTL (**top**) and all-JJTL (**bottom**) obtained by replacing each geometric inductor with a single Josephson junction.

The circuit shown in Figure 1 is a 1-to-4 splitter built from four kinetic-inductance control keys (KICKs) connected to a single Josephson junction. The input line and each of the four output branches is implemented as an all-Josephson-junction transmission line (all-JJTL)—a design where every geometric inductor of a conventional JTL is replaced by a single Josephson junction (Figure 1b). The standard parameters of each all-JJTL cell are: grounded junctions $J_{g}$ with $A_{g}=1$, connecting junctions $J_{con}$ with $A_{con}=0.7$, and bias current $i_{b}=I_{b}/I_{c,\mathrm{ref}}=0.75$. The KICKs allow the propagation of pulses into each line to be independently enabled or blocked by tuning their kinetic inductance.

**Figure 2:**
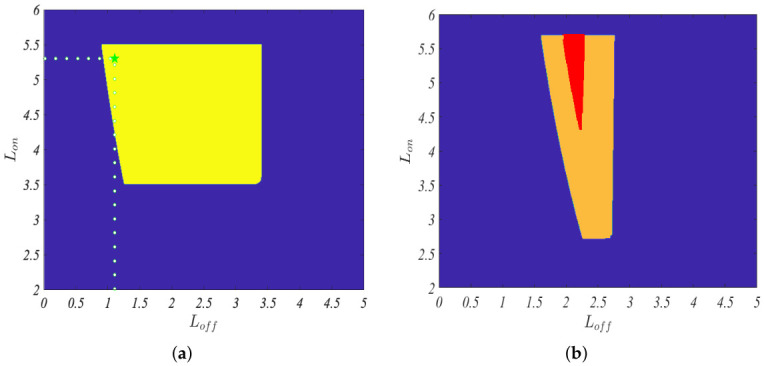
(**a**) Parameter map of ($L_{off}$, $L_{on}$). Using inductance values for enabling/disabling pulse transmission from the yellow region will not disrupt the operational mode of the other lines; the blue region indicates operational failure. The junction parameters are: $J_{con1}$ with A=0.7, $J_{g}$ with A=1, and each of the four $J_{con2}$ junctions with A=0.3. Bias currents: $i_{b1}=1$; $i_{b2}=0.95$. The bias current $i_{b1}=1$ is applied to the common node and splits between $J_{g}$ and the four output branches. According to our simulations, $J_{g}$ starts to generate spontaneously only when $i_{b1}\geq 1.42$, so at $i_{b1}=1$ the junction remains stably superconducting with a comfortable static margin. A five-pointed star marks the point $\left(L_{off},L_{on}\right)=\left(1.1,\;5.3\right)$, which exhibits the largest separation between the blocking and transmitting inductance values while remaining inside the operational region, thereby providing a safe margin for practical implementation. (**b**) Orange: parameter map of valid ($L_{off}$, $L_{on}$) for $A_{con2}=0.45$. Red: parameter map of valid ($L_{off}$, $L_{on}$) for $A_{con2}=0.55$. An increase in the critical current of the connecting junctions $J_{con2}$ leads to a narrowing of the operational parameter region.

The inductance values were selected so that each output line could be controlled independently of the others without affecting the dynamics of the processes occurring within them. Here, $L_{on}$ denotes the inductance value at which pulses pass through to the output line, while $L_{off}$ denotes the value at which pulses are blocked; both are given in units of $L_{J}$. Figure 2a presents a parameter map for possible inductance values. The yellow region indicates pairs ($L_{off}$, $L_{on}$) that ensure correct operation of the splitter scheme, while the blue region corresponds to operational failure. Each logical “1” is generated by exciting the input all-JJTL with a Gaussian current pulse, which launches a single SFQ-like 2π phase-transfer event that reaches the cell, and the output is classified through the transferred flux, i.e., from the number of 2π phase slips of the output junction ($\int V\;dt=\Phi_{0}$ per event) within the detection window. All possible combinations of enabling/disabling the output lines were verified. An increase in the critical current of the connecting Josephson junctions, $J_{con}$, leads to a narrowing of the operational parameter region.

The splitter is the most branched cell in the library—four output lines share a common node—and its operational region is correspondingly the narrowest of those computed here. We therefore use it as a representative and presumably demanding test case for the robustness of the scheme, without claiming that it constitutes a rigorous bound on the margins of the remaining gates with respect to every individual parameter. At the operating point marked in Figure 2, the individual junction critical currents can be varied over wide ranges without disrupting correct operation: $J_{con1}$ tolerates +41/−60%, $J_{g1}$ tolerates +56/−36% and $J_{con2}$ tolerates +60/−54%, while the Josephson damping parameters admit variations exceeding 60% in either direction. The bias currents retain correct operation over a spread of at least ±20%. All of these margins meet or exceed the ±20% criterion commonly adopted as an indicator of robust operation in superconducting digital circuits.

### 3.3. AND/OR

**Figure 3:**
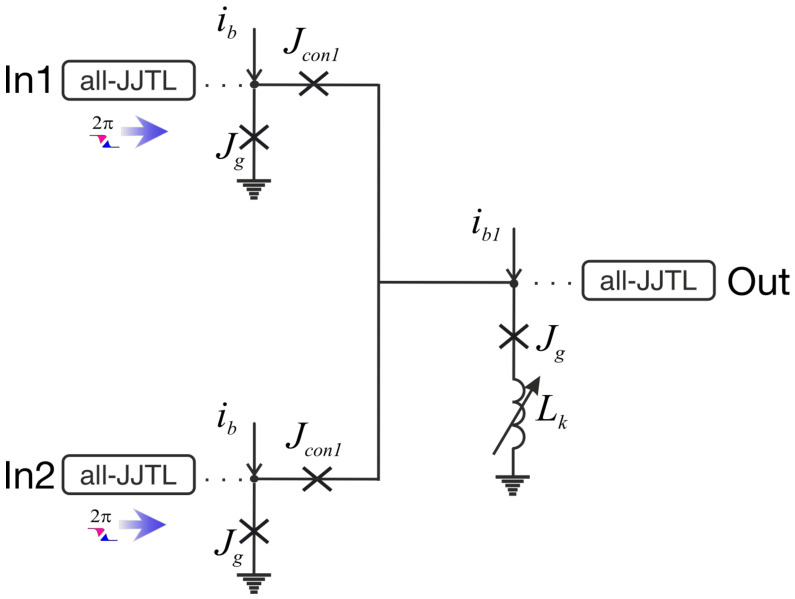
Circuit diagram of a cell with controlled kinetic inductance for switching the circuit’s operation mode between AND and OR logic gates. $J_{con1}$: A=0.3. $i_{b1}=1.2$.

Utilizing kinetic inductance, it is possible to create a logic cell that operates as an AND (for low values of inductance $L_{k}$) or an OR (for high values of inductance $L_{k}$) gate (see Figure 3). For low inductance values ($L_{k}/L_{J}<4.68$), the inductor behaves as a weak barrier: a single input pulse cannot supply sufficient current to trigger the output junction $J_{g1}$. Only when two pulses arrive synchronously do their currents add constructively, overcoming the barrier and switching $J_{g1}$; the cell thus operates as an AND gate. At higher inductances ($L_{k}/L_{J}\in \left[4.68,\;5.71\right]$), the energy storage capability of the kinetic inductor increases considerably. A single pulse now deposits energy in the kinetic inductance (the kinetic energy of the superconducting condensate), and the subsequent release of this stored energy provides an additional current surge that drives $J_{g1}$ well above its critical value. In this regime the cell fires upon receiving any input, behaving as an OR gate. A further increase of $L_{k}/L_{J}$ beyond the value 5.71 leads to the formation of several stable states and gives rise to other complex dynamics.

The AND operation relies on the constructive summation of the currents of two input pulses arriving at the output junction $J_{g1}$: only when both pulses overlap in time does the combined current exceed the switching threshold set by the kinetic inductance. The gate is therefore intrinsically sensitive to the temporal skew Δt between the two inputs. We quantify this tolerance as the largest skew max|Δt| for which the gate still produces exactly one output pulse, obtained by bisection for each parameter value.

To relate the normalised time τ to physical units we adopt a characteristic Josephson plasma frequency $f_{p}=40\;\mathrm{GHz}$, giving a time unit $t_{0}=1/\left(2\pi f_{p}\right)\approx 4$ ps. At the nominal parameters, with $L_{k}$ set to the centre of the corresponding operating window, both modes tolerate an input skew of the order of max|Δt|≈12 (norm.), i.e., ≈50 ps. Notably, the OR mode switches only about three normalised units (≈12 ps) faster than the AND mode, indicating that the kinetic inductance affects the switching dynamics only marginally. In both cases the tolerated skew is comfortably above the timing jitter expected in SFQ circuitry, confirming that the synchronisation requirement is not a practical limitation.

### 3.4. XOR/OR

**Figure 4:**
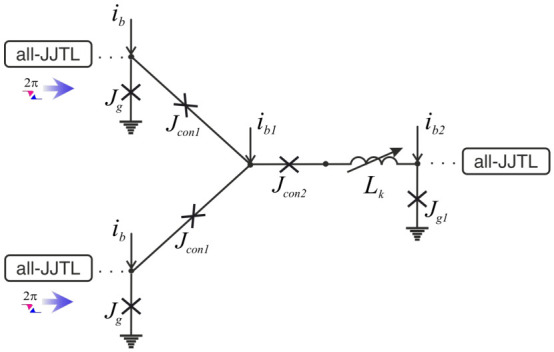
Circuit diagram of a cell with controlled kinetic inductance for switching the circuit’s operation mode between XOR and OR logic gates. $J_{con1}$: A=0.6. $J_{con2}$: A=0.75. $J_{g1}$: A=0.25. $i_{b1}=0.6$, $i_{b2}=0.25$.

The addition of kinetic inductance enables the cell to function as an XOR gate at $L_{k}/L_{J}\in \left[1.32,\;1.85\right]$ and as an OR gate at $L_{k}/L_{J}>1.85$ (see Figure 4).

In this cell the junction $J_{con2}$ acts as a coincidence-triggered blocking element: a 2π phase slip of $J_{con2}$ disrupts the propagating soliton-like pulse and prevents it from reaching the output. Because the current driven through this branch scales inversely with the series inductance, $i∼\Delta \varphi /(L_{k}/L_{J})$, the value of $L_{k}/L_{J}$ sets the effective threshold for switching $J_{con2}$. For low inductance values ($L_{k}/L_{J}\in \left[1.32,\;1.85\right]$ in normalized units), the current delivered to $J_{con2}$ by a single input pulse stays below its critical value, so $J_{con2}$ remains superconducting and the pulse propagates to the output. When two pulses arrive synchronously, the contributions from the two $J_{con1}$ junctions add at the common node, the current through $J_{con2}$ exceeds its critical value, and $J_{con2}$ undergoes a 2π slip that halts the soliton. The output thus fires for exactly one active input and is suppressed for two coincident inputs, so the cell operates as an XOR gate. For higher inductances ($L_{k}/L_{J}\in \left[1.86,\;4.15\right]$), the series kinetic inductance limits the current reaching $J_{con2}$ below its critical value even for two coincident pulses. The junction $J_{con2}$ can therefore no longer switch, the blocking mechanism is disabled, and every input—single or coincident—propagates to the output. In this regime the cell operates as an OR gate.

**Figure 5:**
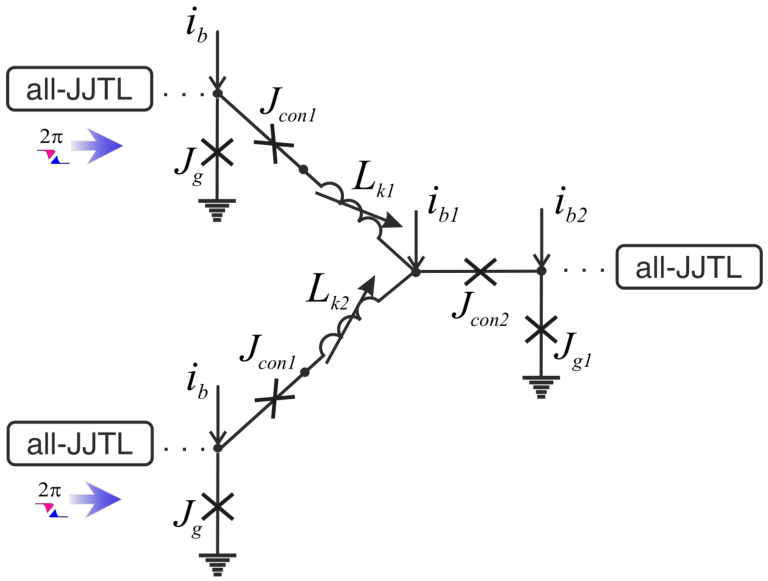
Extended XOR cell with two tunable kinetic inductances, reconfigurable to realise the asymmetric functions $\bar{A}\wedge B$ and, by symmetry, $\bar{B}\wedge A$. $J_{con1}$: A=0.9. $J_{con2}$: A=0.75. $J_{g1}$: A=0.25. $i_{b1}=0.45$, $i_{b2}=0.25$.

We also demonstrate that the functionality of the XOR gate can be further enriched by introducing two additional kinetic inductances into the circuit. The circuit in Figure 5 can be reconfigured to realise the asymmetric function $\bar{A}\wedge B$; specifically, this requires setting $L_{k1}/L_{J}=2.5$ and $L_{k2}/L_{J}=0.5$ (the mirrored function $\bar{B}\wedge A$ is obtained analogously by swapping inductance settings). This transformation is achieved solely by tuning the kinetic inductance values, without any changes to the active Josephson junctions or the addition of new bias lines. The ability to realise the non-commutative function is particularly attractive for neuromorphic or bio-inspired spiking neural networks. In such systems, the directionality of synaptic connections plays a fundamental role: the postsynaptic neuron *C* may be required to respond to a spike from the neuron *B* only when the presynaptic neuron *A* has not fired recently, implementing a form of gated inhibition or a directional receptive field. This operation naturally arises in circuits mimicking spike-timing-dependent plasticity (STDP), where the relative order of pre- and postsynaptic events determines whether a synapse is potentiated or depressed, or in Winner-Take-All architectures [45,46,47,48], where the activity of one input must selectively suppress or gate another.

## 4. Reconfigurable Logic Basis and Universal Programmable Cell

Building upon the fundamental AND/OR and XOR/OR gates with controllable kinetic inductance, we propose a novel architecture for a universal programmable logic cell (UPC). While the individual reconfigurable gates do not constitute a complete logical basis, the incorporation of a constant logical “1” enables the implementation of inversion functionality through XOR operations, thereby forming a universal set.

**Figure 6:**
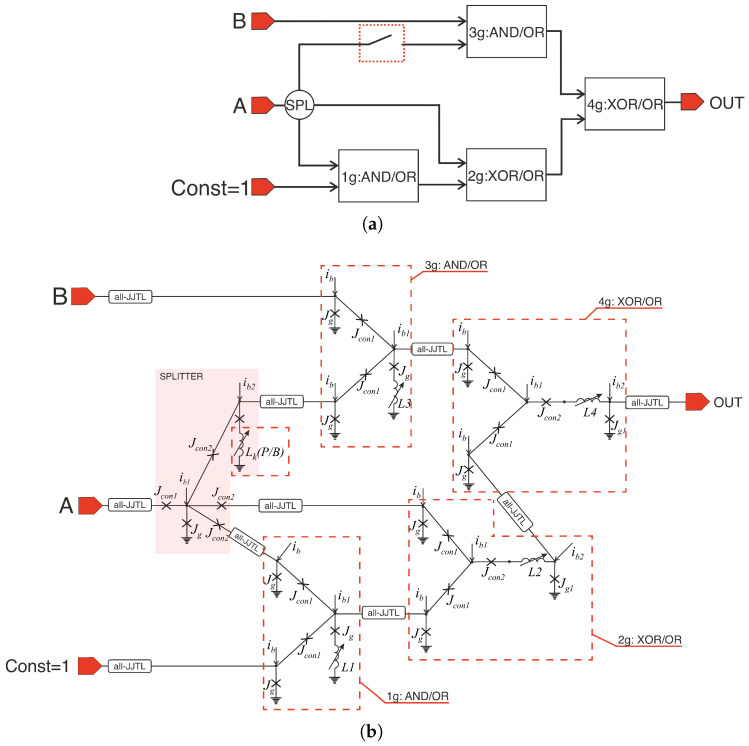
(**a**) Architecture of the universal programmable logic cell based on reconfigurable all-JJ gates. The circuit consists of four reconfigurable gates (XOR/OR and AND/OR types) and utilizes a constant logical “1” for inversion operations. A splitter at input *A* distributes the signal, and a tunable kinetic inductance in one of its branches acts as an additional programmable element with two states: pass and block. Together with the four gates, the state of this inductance determines which Boolean function is realised. (**b**) Circuit-level schematic of the same cell at the Josephson-junction level, showing the individual junctions, bias currents and KICK elements. The parameters of the splitter and of all four gates are listed in Table 1.

The proposed architecture, illustrated in Figure 6, represents an analogue of a Look-Up Table (LUT) commonly used in FPGA architectures. However, instead of storing precomputed truth tables in memory, our approach implements a configurable logical basis where the functionality is defined by the operational modes of the kinetic inductance gates. This provides dynamic reconfigurability at the hardware level while maintaining the performance benefits of superconducting electronics.

To identify the minimal circuit capable of realising all sixteen two-input Boolean functions, we performed an exhaustive depth-first search over feed-forward circuits built from reconfigurable AND/OR and XOR/OR gates. The available signal sources are the primary inputs *A* and *B*, a constant logical “1”, and the outputs of all previously placed gates. For a fixed number of elements *N*, the search recursively constructs a circuit by assigning to each element two input signals from the admissible set and a gate type, either AND/OR or XOR/OR. Once a complete topology is assembled, its functional coverage is evaluated: for every combination of the configuration bits (there are $2^{N}$ of them, each selecting the operating mode of one element), the output is computed for the four input patterns (A,B)=(0,0),(0,1),(1,0),(1,1), and the set of distinct truth tables so obtained forms the coverage mask of the topology. A topology is universal if its mask contains all sixteen functions.

The tunable kinetic-inductance key need not be treated as a separate primitive in this search. An XOR/OR gate whose two inputs are driven by the same signal *S* realises S⊕S=0 in the XOR mode and S∨S=S in the OR mode, i.e., it is a pure block/pass element; it requires no Josephson-junction circuitry and no additional bias line, and is implemented by a single tunable kinetic inductance placed in one branch of the splitter that fans out *S*. The KICK is thus a limiting case of an XOR/OR gate, so a search over the pure gate basis automatically covers all mixed gate-plus-key architectures, and the quantity to be minimised is simply the total number *N* of configurable elements.

A lower bound on *N* follows from counting: a topology with *N* configurable elements admits $2^{N}$ configurations and therefore realises at most $2^{N}$ distinct truth tables, so N≥4 is necessary; moreover, at N=4 the map from configurations to functions would have to be a bijection, with no two of the sixteen configurations collapsing onto the same function. The enumeration is made tractable by three reductions: since AND, OR and XOR are commutative, the two inputs of an element may be ordered without loss of generality; topologies containing an element whose output feeds no later element are redundant, as every element must be an ancestor of the output; and an AND/OR gate with both inputs on the same signal acts as a buffer in both modes and is discarded, in contrast to the degenerate XOR/OR gate discussed above. Together these rules reduce the enumeration at N=5 from $6.35\times 10^{6}$ to $2.82\times 10^{5}$ inequivalent topologies, exhausted within seconds on a single core.

The search finds no universal topology for N≤4. The best four-element circuit covers only twelve of the sixteen functions, failing to reach XOR, NAND, XNOR and B⇒A; once the four configuration bits have been spent on the remaining twelve functions, no parity-type stage is left to generate these. Five elements are therefore necessary, and at N=5 the search returns 74 universal topologies, so five is also sufficient and hence minimal. Ranking these solutions by physical cost, the optimum is attained by four full reconfigurable gates together with one degenerate element, i.e., one kinetic-inductance key and a single splitter; twelve topologies attain it, all equivalent under the A↔B symmetry and under gate reordering. No universal five-element cell built from three gates and two keys exists, so the key count cannot be traded further against the gate count.

The resulting optimal cell is shown in Figure 6: two XOR/OR gates, two AND/OR gates, and a splitter at input *A* distributing the signal into three lines, one of which carries a tunable kinetic inductance. The thirty-two hardware states of its five tunable kinetic-inductance elements reproduce the full set of sixteen two-input Boolean functions.

**Table 1:** Parameters of the splitter and the four reconfigurable gates of the universal programmable cell (Figure 6). A dash denotes an element absent in the given cell.

Cell	Type	$A_{con1}$	$A_{con2}$	$A_{g1}$	$i_{b1}$	$i_{b2}$
Splitter	1-to-3	0.7	0.3	1.0	1.0	0.95
2 g, 4 g	XOR/OR	0.6	0.75	0.25	0.6	0.25
1 g, 3 g	AND/OR	0.3	–	1.0	1.2	–

The programmable cell is configured by setting the state of five tunable kinetic inductances: one in the splitter (pass or block) and one in each of the four gates, which determines their operational mode (AND/OR or XOR/OR). This configurability enables the same physical circuit to implement diverse logical functions, including but not limited to: AND, OR, XOR, NAND, NOR, XNOR, and various implication functions.

Potential applications of this technology include:

- Dynamically reconfigurable superconducting processors for adaptive computing;
- Energy-efficient neuromorphic computing systems requiring programmable connectivity;
- Quantum-classical interface circuits with adaptable signal processing capabilities.

The universal cell described earlier requires an external constant “1” line to implement inversion within the logic fabric. A self-contained variant can be obtained by placing a single D-flip-flop with complementary outputs on one of the primary inputs, for instance, on input *A*. The flip-flop stores the logical value of *A* and provides both the true (*A*) and the inverted ($\bar{A}$) version of this variable; these are fed into an additional AND/OR gate configured as an OR gate, whose output serves as the internal constant “1”.

The pulse-level operation of this arrangement is as follows, and is fully consistent with the convention of Section 2. A read-out flip-flop of the RSFQ DRO/NDRO type with complementary outputs emits, upon each clock event, exactly one SFQ pulse: on the true output if the stored value is “1” and on the inverted output if it is “0”. Merging the two outputs in an OR-configured gate therefore yields exactly one output pulse per clock event, irrespective of the stored value. The constant “1” is thus not a maintained static level but a pulse delivered once per logic-evaluation cycle, exactly as required by the definition of a logical “1” adopted in Section 2. Its latency—the flip-flop read-out delay plus the propagation delay of the merging gate—is fixed and known, and is compensated by matched all-JJTL padding on the *A* and *B* paths, so that the constant-“1” pulse enters the first logic stage within the same timing window as the primary inputs.

This variant trades external wiring for local circuitry: the flip-flop and the merging gate increase the junction count, the routing and the area of the cell. The availability of complementary-output D-flip-flops in standard superconducting libraries guarantees process compatibility—no new fabrication process is required—but not the absence of circuit-level overhead. The gain is autonomy: no constant-“1” line has to be distributed from outside, which improves regularity in large arrays at the cost of a larger individual cell.

**Figure 7:**
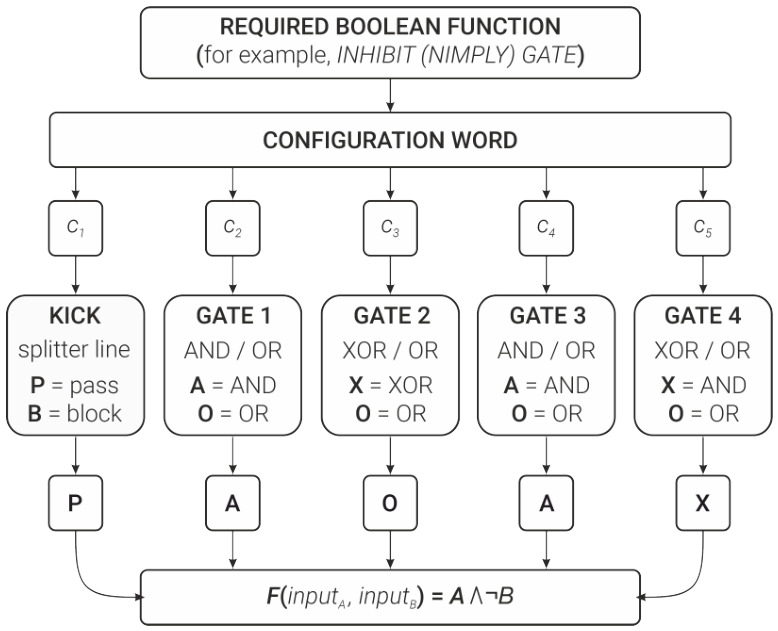
Encoding of the universal programmable cell configuration into a five-letter word $c_{1}c_{2}c_{3}c_{4}c_{5}$: one letter per tunable kinetic-inductance element (P/B for the splitter KICK, A/O for the AND/OR gates, X/O for the XOR/OR gates). Each of the $2^{5}=32$ words selects one Boolean function *F* of the inputs *A* and *B*, together covering all sixteen two-input functions (Table 2).

For clarity, we encode each configuration of the universal programmable cell by a five-letter word corresponding to the operating modes of a kinetic inductance and gates 1–4 in Figure 6. For the XOR/OR gates, the letter (X) denotes the XOR mode and (O) denotes the OR mode; for the AND/OR gates, the letter (A) denotes the AND mode and (O) denotes the OR mode; for the tunable kinetic inductance in the splitter line, the letter (P) denotes the pass state (high $L_{k}$) and (B) denotes the block state (low $L_{k}$). Thus a configuration word specifies a particular hardware state of the five tunable kinetic-inductance elements. This representation is useful because it makes the programmable cell closer to a small reconfigurable logic fabric than to a conventional memory-based LUT: the function is selected by physical operating modes of gates rather than by reading a stored truth table. As summarised in Figure 7, sweeping all $2^{5}=32$ configuration words produces the full set of sixteen two-input Boolean functions.

**Table 2:** Truth table of all two-input Boolean functions and their relevance to bio-inspired spiking neural networks. Functions are grouped by symmetry and potential use in neuromorphic circuits.

Inputs (A,B)				Function	Configured Mode	Neuromorphic Relevance
(0,0)	(0,1)	(1,0)	(1,1)			
Constant functions						
0	0	0	0	FALSE	BAXAX, BAXAO	Complete inhibition (silent)
1	1	1	1	TRUE	BOOAX, BOOAO, BOOOO, POXOO, POOAO, POOOO	Tonic excitation
Symmetric functions						
0	0	0	1	AND	PAXAX, PAXAO	Coincidence detection
0	1	1	1	OR	BAOOO, PAXOX, PAXOO, PAOOO	Input integration
0	1	1	0	XOR	BAOOX	Coincidence/ anti-coincidence (STDP)
1	1	1	0	NAND	POOAX	Universal gate
1	0	0	0	NOR	POOOX	Universal gate
1	0	0	1	XNOR	BOXOX	Equivalence detection
Projection and inversion						
0	0	1	1	*A*	BAOAX, BAOAO, PAOAO	Signal pass-through
0	1	0	1	*B*	BAXOX, BAXOO	Signal pass-through
1	1	0	0	NOT *A*	BOXAX, BOXAO	Inversion
1	0	1	0	NOT *B*	BOOOX	Inversion
Asymmetric (inhibition/implication)						
0	0	1	0	A∧¬B	PAOAX	Directional gating, lateral inhibition
0	1	0	0	B∧¬A	PAOOX	Directional gating, lateral inhibition
1	0	1	1	B⇒A	POXOX	Conditional activation
1	1	0	1	A⇒B	BOXOO, POXAX, POXAO	Conditional activation

## 5. Kinetic Inductances in Spiking Neural Network Elements

**Figure 8:**
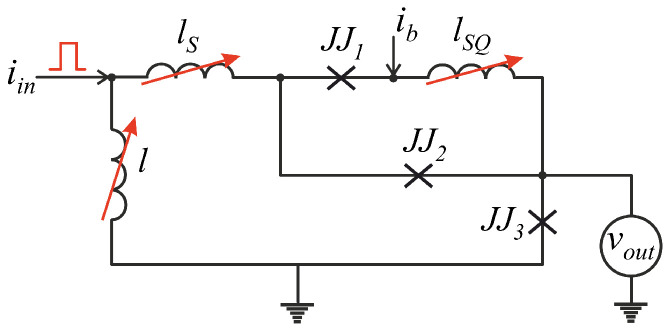
Model of a superconducting bio-inspired neuron’s soma adapted from [53]. Conventional circuit symbols for inductors, with red arrows drawn on them, denote tunable kinetic-inductance elements.

**Figure 9:**
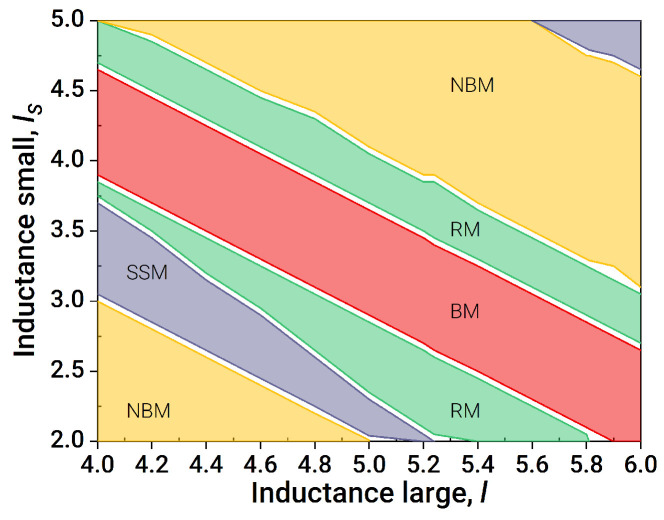
Parameter domain of the superconducting bio-inspired neuron in the *l*–$l_{S}$ inductance plane. The labelled regions correspond to regular mode (RM), steady-state mode (SSM), bursting mode (BM), and non-biological mode (NBM). The fixed parameters are $l_{SQ}=l+l_{S}$, $I_{b}/I_{C3}=2.5$, Josephson damping parameters $\alpha_{1,2,3}=0.78$ (in [53] they were designated by $\Gamma_{1,2,3}$), $I_{C1}/I_{C3}=1$ and $I_{C2}/I_{C3}=0.97$ ($I_{C3}$ used as the normalization reference).

Kinetic inductance can also be used in superconducting neuromorphic circuits [49,50,51,52], in particular in bio-inspired spiking neural networks [50,53]. This neuromorphic extension is consistent with the broader view that superconducting electronics can provide ultrafast, low-dissipation hardware for spike-based computation [54], while Josephson junctions naturally support neuron-like threshold, spiking, and bursting dynamics [49,55,56]. In such systems, the main functional elements are the neuron’s soma, the axon-like transmission line, and the synaptic connection. Replacing geometric inductances by tunable kinetic inductances can reduce the occupied area, improve scalability, and suppress parasitic magnetic coupling associated with large inductive loops. At the same time, kinetic inductance provides an additional control parameter for tuning thresholds, propagation delays, and dynamical regimes of spiking elements as demonstrated in Figure 8 and Figure 9.

At the same time the neuromorphic relevance of the universal programmable cell should not be understood as a direct one-to-one mapping between Boolean gates and biological neurons. Instead, the cell provides a compact library of local event-driven operations that are repeatedly needed in spiking neural hardware. In such systems, spikes must be combined, suppressed, delayed, routed, and conditionally transmitted. Boolean functions naturally describe these local operations at the level of pulse presence or absence within a timing window. For example, the AND gate implements coincidence detection, the OR gate implements input integration, the XOR gate implements anti-coincidence or mismatch detection, and asymmetric functions such as $\left(A\wedge \bar{B}\right)$ implement inhibition or conditional routing.

This interpretation also explains why reconfigurability is especially natural in neuromorphic applications. Unlike conventional fixed digital logic, neuromorphic circuits often require changes in effective connectivity, receptive windows, inhibition patterns, and routing rules. A kinetic-inductance-controlled cell can therefore serve as a local programmable element whose function can be adapted without redesigning the circuit layout. In this sense, the same universal cell bridges conventional Boolean logic and spike-based neuromorphic processing.

### 5.1. Neuromorphic Applications of the Universal Programmable Cell

For neuromorphic use it is not necessary to implement all sixteen Boolean functions: a small subset already covers the local, event-driven operations that recur in spiking hardware, which simplifies configuration and fabrication. Reusing the correspondence introduced above (AND for coincidence detection, OR for input integration, XOR for anti-coincidence or mismatch detection, and the asymmetric $A\wedge \bar{B}$ for inhibition or conditional gating), these primitives can be extended in time by driving a cell with delayed copies of its inputs. Applied to two time-shifted copies of one signal, A∧B emulates the coincidence window associated with long-term potentiation (LTP), while $A\wedge \bar{B}$ produces the complementary cut-off associated with long-term depression (LTD); cascading the two gives the potentiation/depression asymmetry of spike-timing-dependent plasticity (STDP). Cross-coupling the inhibitory inputs of several cells implements winner-take-all competition and lateral inhibition [45,46,47,48], feeding a cell’s output back to its own inhibitory input through a delay line emulates a refractory period, and linking several such blocks through delay lines gives access to rhythmic and bursting network activity.

These correspondences are stated as design motivation, not as modelled results. What the present simulations establish directly is that each cell reproduces its target Boolean truth table under the operating criteria of Section 2, and that all sixteen functions are available within one reconfigurable block. Whether a given configuration reproduces a specific biological mechanism quantitatively, with the correct time constants, thresholds and plasticity curves, is left to future work on complete spiking-network circuits.

Taken together, these operations suggest a concrete way of embedding the proposed cells into a larger spiking system. Because the cells are built in the all-JJ framework and communicate through the same SFQ-like pulses as existing superconducting somas, synapses and Josephson transmission lines, they can be inserted directly into the pulse-routing fabric between neurons without a change in signal representation: the soma integrates inputs and fires, the transmission lines set the propagation delays that define the timing windows, and a UPC placed at a dendritic or synaptic node performs the local event operation. The non-volatile, locally field-programmed KICK adds a second, slower timescale on top of this: fast pulse logic carries out the event-driven computation within a clock period, while reconfiguration of the kinetic-inductance states rewires the effective connectivity and gating rules of the array between tasks, in the spirit of a cryogenic reconfigurable fabric.

The primitives that map most naturally onto this hardware are coincidence detection (AND), temporal integration (OR), mismatch or novelty detection (XOR), and gated inhibition or directional routing ($A\wedge \bar{B}$), together with the refractory blocking and winner-take-all behaviour obtained by adding delayed feedback and cross-inhibition. Local learning rules of the spike-timing-dependent type fit the same structure: the potentiating branch of STDP corresponds to A∧B applied to a signal and its delayed copy, and the depressing branch to $A\wedge \bar{B}$, so that a pair of configured cells reproduces the two halves of an STDP update. Combined with a plastic weight element, for instance, a superconducting STDP synapse of the configuration described in Ref. [51] or a self-training superconducting architecture such as that of Ref. [52], the UPC supplies the surrounding coincidence, gating and routing logic, while the synapse stores the adapted weight. We present these as concrete integration pathways rather than demonstrated network results. A quantitative study of coupled multi-cell circuits, including timing margins and plasticity curves, is the natural next step.

### 5.2. Kinetic Inductance in the Neuron Soma

The neuron soma is the part of a spiking element where the input stimulus is integrated and converted into an output voltage spike. Earlier, we proposed a superconducting bio-inspired neuron intended for operation in spiking neuromorphic networks [57] that emulate basic patterns of biological neural activity. In that design, the dynamical regime of the neuron is controlled by the circuit parameters and by the external bias current $i_{b}$. Since the generation of an output voltage spike is determined by the phase dynamics of the Josephson circuit, replacing selected geometric inductances by kinetic inductances offers a direct way to tune the soma dynamics after fabrication.

**Figure 10:**
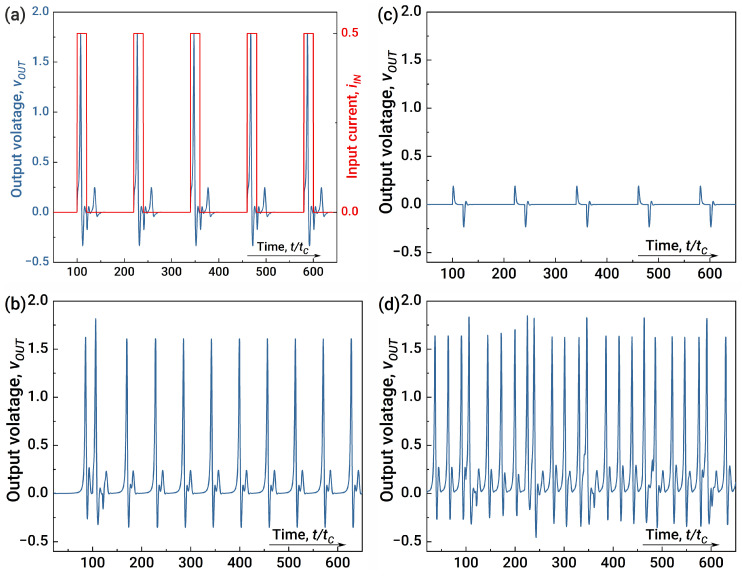
Representative output-voltage traces of the superconducting bio-inspired neuron in different operating regimes: (**a**) regular mode, (**b**) bursting mode, (**c**) steady-state mode, and (**d**) non-biological mode.

Some of the present authors also contributed to Ref. [53], and the soma circuit considered here builds on the three-junction bio-inspired neuron introduced in that work. Its schematic in Figure 8 is adapted from that neuron with partial modifications. As a starting point we adopt the parameters that in Ref. [53] demonstrated regular-mode operation of the soma, and we then examine how the neuron’s operating regimes evolve once the selected geometric inductances *l*, $l_{S}$ and $l_{SQ}$ (=$l+l_{S}$) are realised as tunable kinetic inductances. The reference point was based on the following parameter values: $I_{b}/I_{C3}=2.5$, Josephson damping parameters $\alpha_{1,2,3}=0.78$ (in [53] they were designated by $\Gamma_{1,2,3}$), $I_{C1}/I_{C3}=1$ and $I_{C2}/I_{C3}=0.97$. Note that a sequence of rectangular pulses at the input with a total level $A_{in}=0.5$, period $T_{in}=120\cdot t_{p}$ and duration of one pulse $\tau_{in}=20\cdot t_{p}$ was used as an external stimulus (here $t_{p}$ is the inverse plasma frequency, defined in Section 2). Varying the indicated kinetic inductances with the other parameters fixed revealed three basic modes of neuron operation: regular mode (RM), steady state mode (SSM) and bursting mode (BM). In Figure 9 all the mentioned modes are marked with the corresponding designations. A separate area is occupied by the so-called non-biological mode of operation (NBM), which is characterised by continuous generation of spikes without any external influence (no input stimulus signal) and pattern activity. Typical spike-generation patterns of the neuron in all four regimes are shown in Figure 10.

## 6. Discussion and Conclusions

We have presented a compact set of basic cells for reconfigurable superconducting kinemonics, where kinetic inductance is used not only as a replacement for bulky geometric inductors but also as a functional control parameter. The proposed cells include a controllable splitter and two reconfigurable logic gates, AND/OR and XOR/OR. In each case, changing the kinetic inductance modifies the propagation of SFQ-like pulses and selects the operating mode of the same physical circuit.

Combining these elementary cells, we constructed a universal programmable logic cell consisting of four reconfigurable gates and a single tunable kinetic-inductance key. An exhaustive search established that this is the minimal number of configurable elements able to realise all sixteen two-input Boolean functions, so that the cell provides a superconducting analogue of a look-up table. In contrast to a conventional LUT, however, the functionality is not stored as a truth table in memory; it is defined by the selected modes of the kinetic-inductance-controlled gates. This makes the proposed architecture attractive for dense superconducting circuits where functional density, compactness, and post-fabrication tunability are equally important.

We also considered the role of kinetic inductance in superconducting spiking-neuron elements. In the neuron soma, replacing selected geometric inductances by kinetic inductances allows one to tune the dynamical regime of the circuit and to shift the boundaries between regular spiking, steady-state, bursting, and self-oscillatory operation. Thus, the same design principle can be used both in reconfigurable digital logic and in bio-inspired superconducting neuromorphic circuits.

The main practical advantage of this approach is the simultaneous reduction of footprint and parasitic magnetic coupling. Smaller inductive loops reduce unwanted flux cross-talk, while tunable kinetic inductance provides an additional degree of freedom for compensating fabrication spread and adjusting the circuit after fabrication. In physical units, for a representative normalization current $I_{c,\mathrm{ref}}=100\;$µA, the unit of normalized inductance corresponds to $\Phi_{0}/\left(2\pi I_{c,\mathrm{ref}}\right)\approx 3.3$ pH, so all operating values of $L_{k}$ used in this work lie between a few and about twenty picohenry. Sheet kinetic inductances measured in thin NbN strips reach tens to a hundred picohenry per square [6,10,58], so the required values correspond to strips well below one micrometre in length even for moderately disordered films. The present results are based on numerical modelling; the experimental outlook is summarized at the end of this section.

The universal programmable logic cell contains 140 Josephson junctions in total, of which 30 are used in the logic blocks and approximately 110 in the interconnection lines. All logic blocks are interfaced via all-JJTL to completely eliminate static coupling between them. For the sole purpose of indicating the junction-area scale, we adopt the MIT-LL SIS tunnel-junction technology (0.0625µm^2^ per junction for a 0.25µm^2^ diameter) [4] or, alternatively, the NbAl-Al-AlNb bridge technology, which needs no external shunting (0.05µm^2^ per junction) [59]. On this basis the 140 junctions correspond to 8.75µm^2^ and 7.0µm^2^, respectively. These figures are lower bounds rather than complete circuit footprints: they exclude design-rule spacing, wiring, vias, bias distribution, and junction shunts where required, and a complete design-rule-based layout would be needed for a technology-level footprint estimate. In particular, no nominal area is assigned here to a magnetic KICK, because its layout depends on the multilayer sheet inductance, the contact requirements, and the magnetic-programming scheme, none of which is yet fixed for the specific Nb/Co/Nb/Co/Nb/Al structure. For the same reason, the preceding NbN-based conversion of the required inductances into submicrometre dimensions is illustrative only and concerns generic high-kinetic-inductance nanostrips; it does not determine the physical footprint of the proposed magnetic KICK, whose sheet inductance and minimum practical dimensions may differ substantially.

Where a device-count comparison is meaningful, it is drawn only against other Josephson-junction implementations, for which the junction number is the natural common measure. A functionally equivalent two-input superconducting LUT (four truth-table entries), based on a two-dimensional NDRO memory-cell array with an SFQ decoder, uses 313 Josephson junctions [60], against the 140 of the present cell. That design is a complete, fabricated and experimentally tested layout occupying 0.25 mm^2^, whereas the figure quoted above for our cell is a lower-bound active-device area; the two areas are therefore not directly comparable, and it is the junction count—independent of design rules and wiring—that provides the homogeneous comparison. The difference reflects architecture rather than process: in the memory-based LUT the sixteen functions are stored in an addressed cell array with its decoder, whereas in the present cell the function is set by the operating modes of the gates. That implementation also relies on geometric inductances in each memory cell, extracted from the layout, whereas the present cells follow the all-JJ approach in which geometric inductors are replaced by Josephson junctions, the only inductive elements being the tunable kinetic-inductance keys.

Cryogenic CMOS circuits can operate at low temperatures [61,62,63]. The specific motivation for the present superconducting implementation is its native compatibility with SFQ pulse processing and superconducting-circuit technology, picosecond-scale operation, low dissipation, and the associated system-level cryogenic power budget, while direct comparison of the total chip area is premature at this stage of development.

All results reported here are obtained from deterministic RCSJ equations; thermal and other stochastic current sources are not included. In a stochastic extension the resistive branches would contribute Johnson–Nyquist noise-current terms [64,65]. Near a switching boundary such fluctuations can cause premature or missed switching, broaden the transitions between the logic modes, increase pulse-timing jitter, and activate transitions between coexisting dynamical states; similar effects may blur the boundaries between the neuron-soma regimes of Figure 9. The domains reported here should therefore be regarded as zero-noise reference regions, with operating points selected well inside them; the regions found are comparatively wide, which is expected to ease the noise requirement. Quantitative bit-error rates and noise-induced shifts of the boundaries require stochastic Monte Carlo simulation and lie beyond the scope of the present circuit-concept study.

## Data Availability

The original contributions presented in this study are included in the article. Further inquiries can be directed to the corresponding author.
